# Post-mortem computed tomography angiography using left ventricle cardiac puncture: A whole-body, angiographic approach

**DOI:** 10.1371/journal.pone.0183408

**Published:** 2017-08-21

**Authors:** Yu Shao, Lei Wan, Jianhua Zhang, Zhengdong Li, Ningguo Liu, Ping Huang, Donghua Zou, Yijiu Chen

**Affiliations:** Shanghai Key Laboratory of Forensic Medicine, Shanghai Forensic Service Platform, Institute of Forensic Science, Ministry of Justice, P.R. China, Shanghai, China; University of British Columbia, CANADA

## Abstract

Post-mortem computed tomography (PMCT) and PMCT angiography (PMCTA) are rapidly becoming effective and practical methods in forensic medicine. In this article, the authors introduce a whole-body PMCTA approach involving left ventricle cardiac puncture. This procedure was performed in 9 males and 3 females. PMCT was performed first. Then a biopsy core needle was used for a percutaneous puncture into the left ventricle through the intercostal area under CT guidance. 1000 mL of contrast media (diatrizoate meglumine and normal saline [0.9%] at 1:2 ratio) was injected at a rate of 50 mL/8 s, followed by CT scan. Visualization of systemic arteries was achieved in 11 cases, while only partial visualization was achieved in 1 case, which may have been related to incomplete thawing of the cadaver. PMCTA results revealed no vascular diseases and abnormalities in 10 victims. Among the 10 victims, 4 post-scan autopsies were performed and found no vascular abnormalities, consistent with the PMCTA results. Autopsy of the other 6 victims were refused by the relatives. PMCTA revealed signs of internal carotid artery aneurysm inside the sphenoid sinus in one victim, which was confirmed by autopsy. PMCTA results of another victim showed signs of stenosis and blockage of the distal part of the right vertebral artery and basilar artery. Thromboembolism of those arteries was found during autopsy. Compared with other existing PMCTA methods for examination of vascular injuries and diseases, this technique involves simple procedures, is less time consuming, has lower associated costs, does not require specialized equipment, provides adequate imaging quality, and is suitable for centres not equipped with cardiopulmonary bypass machines or other specialized equipment.

## Introduction

Post-mortem computed tomography (PMCT) is rapidly becoming an effective and practical method in forensic medicine. In general, PMCT has demonstrated adequate sensitivity for the detection of gunshot wounds, mechanical asphyxia, mechanical injury, and drowning [1–3]. However, the accuracy of non-contrast enhanced PMCT for the diagnosis of vascular disease or trauma is low. PMCT angiography (PMCTA), however, enables better visualization of arteries than non-contrast-enhanced PMCT, and permits the evaluation of stenosis and occlusions [4]. It has been reported that the combination of PMCT and PMCTA is very helpful in diagnosing certain fatal injuries and vascular disease [5–7]. Worldwide, several forms of whole-body PMCTA have been described, including whole-body infusion angiographic techniques from Switzerland, and cardiopulmonary resuscitation to establish circulation in Japan [8–12]. In previous research, we reported a case in which aortic rupture was successfully detected by the combination of PMCT and PMCTA using cardiac puncture [4]. Here we introduce a whole-body PMCTA approach involving left ventricle cardiac puncture, which is simple, convenient, and effective for the visualization of arteries.

## Materials and methods

### Materials

A total of 12 adult cadavers (9 male, 3 female), ranging in age from 27 to 75 years, were included in this study. The bodies were of medium body shape and without developmental malformations. According to the medical records, none of the 12 cadavers had previous vascular disease or injury. The post-mortem interval for the 12 bodies was between 2 and 14 days, and all cadavers were frozen and thawed. This study was approved by the Academic Committee of the Institute of Forensic Science, Ministry of Justice, People’s Republic of China. The experimental procedures complied with the relevant protocols of forensic radiology and forensic autopsy. Permission to perform the PMCT and PMCTA were obtained from the relatives of the 12 cadavers. Permission to perform the following autopsy was obtained from the relatives of 6 cadavers (the rest 6 bodies were not dissected). Written informed consents were obtained to publish these case details.

### PMCT and PMCTA

As described in detail previously [4], the entire body was scanned using a 40-slice multi-slice computed tomography system (MSCT; Definition AS, Siemens Medical Solutions, Munich, Germany). Raw data were acquired using the following settings: voltage, 120 kV; current, 240 mAs; collimation, 6.0×1.0 mm. Image reconstruction was achieved at slice thicknesses of 5.0 and 0.625 mm, each with an increment of half the slice thickness. Image review and three-dimensional reconstructions were performed on a computed tomography workstation (Syngo Imaging XS; Siemens Medical Solutions, Germany). Then, during PMCTA, a 14 gauge, 160 mm biopsy core needle (ACN III™, Argon Medical Devices, USA) was used. The entry point, path, and depth of puncture were chosen according to the position of the heart and the distance from the left ventricle to the body surface was measured from the MSCT data. A percutaneous puncture into the left ventricle through the intercostal area was then performed under MSCT guidance. During the puncture process, MSCT scan was performed to ensure the needle maintained the correct path. The drawing of adequate blood was also an indication of proper placement of the needle into the left ventricle. After collecting samples of blood for toxicological analysis, 1000 mL of contrast media (diatrizoate meglumine and normal saline [0.9%] at 1:2 ratio) was manually injected at a rate of 50 mL/8 s. MSCT scan was performed directly after administration of the contrast media solution. Subsequently, an additional 500 mL of contrast media was injected, and MSCT scan was performed again and results were compared.

### Autopsy and other analysis

Traditional autopsy was performed in 6 cases, about one hour after the PMCTA examination. Blood taken by cardiac puncture was sent for toxicological analyses before the contrast agent injection in the PMCTA procedure. During autopsy, external and internal examinations of the body were performed. Histology samples of most of the organs within the cranial, thoracic and abdominal cavity were taken and processed in H&E staining.

## Results

A single PMCT and PMCTA procedure took approximately 30 min to complete.

**Fig 1:**
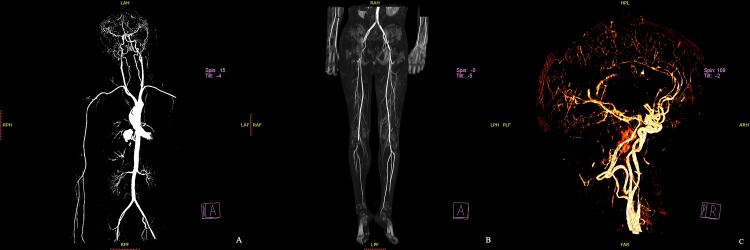
The whole body PMCTA results of a single cadaver.

Left ventricle cardiac puncture was performed successfully in all 12 bodies. 1000mL of contrast agent was injected, following by MSCT scan. The systemic arterial circulatory system could be visualized in cadavers No.1 through No.9, No.11 and No.12, showing a good vascular coverage and image quality (Fig 1). Although blood vessels in the head, neck, and upper limbs could be visualized in cadaver No.10, those below the level of the aortic arch could not. An additional 500 mL (total 1500 mL) of contrast agent was injected and CT scan was performed. Visualization was not significantly different in the 11 cadavers, whereas blood vessels below the aortic arch remained undetectable in cadaver No.10. No vascular abnormalities or pathological changes were found in cadavers No.1 through No.10. Autopsies of the 4 victims also found no vascular diseases or lesions, consistent to the PMCTA results.

**Fig 2:**
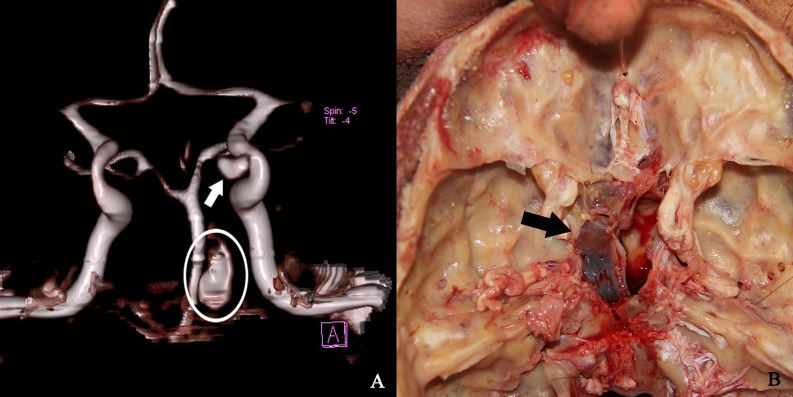
PMCTA, autopsy and histological findings of a single cadaver. A. PMCTA results showed a leakage of the contrast agent from the C3 segment of the left internal carotid artery (arrow) into the adjacent left sphenoid sinus (circle), suggesting an aneurysm. B. An aneurysm inside the left sphenoid sinus (arrow) was confirmed by autopsy, consistent to the PMCTA results.

PMCTA results of the cadaver No.11 revealed that a leakage of contrast agent from the C3 segment of the left internal carotid artery into the adjacent left sphenoid sinus. An aneurysm inside the sphenoid sinus was suspected and was confirmed by the following autopsy (Fig 2).

**Fig 3:**
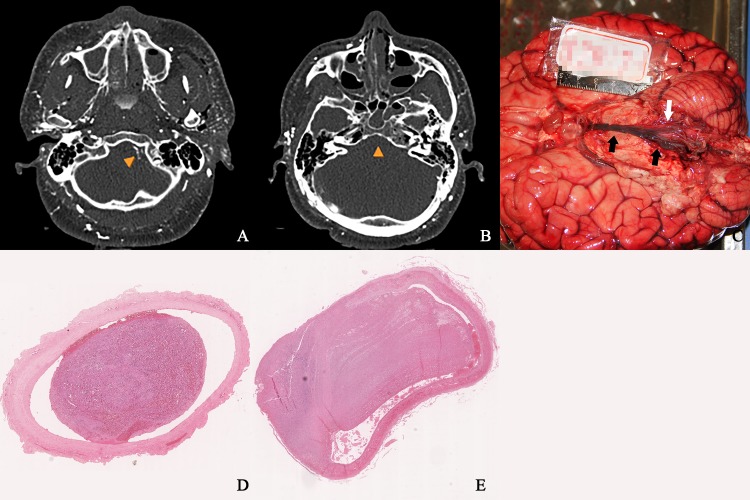
PMCTA and autopsy results of a single cadaver. A. PMCTA results showed no visualization of the distal part of the right vertebral artery, while good demonstration of the left vertebral artery (arrowhead) was obtained. B. PMCTA results showed no visualization of the basilar artery (arrowhead). C. During autopsy, the distal part of the right vertebral artery and basilar artery were found dilated and embolized (black arrows), while the left vertebral artery remained normal (white arrow). D. Histological examination confirming thromboembolism in the distal part of the right vertebral artery. (H&E ×40). E. Histological examination confirming thromboembolism in the basilar artery. (H&E ×20).

PMCTA results of the cadaver No.12 showed no visualization of the distal part of the right vertebral artery and basilar artery, while good demonstration of the left vertebral artery and the circle of Willis were obtained, which was a sign of stenosis and blockage of the vessels. Thromboembolism of those arteries was found during autopsy (Fig 3).

## Discussion

Deaths due to vascular injuries or diseases are commonly observed in forensic practice. Because the systemic vascular system of the human body has widespread distribution and complex anatomical structures, traditional autopsies often only involve examining major blood vessels in conventional anatomical regions (e.g., intracranial, neck, chest, and abdominal cavity). Thus, injuries and diseases that occur in smaller vascular branches, vessels with complex structures, obscured sites, or non-conventional anatomical regions are easily overlooked and lead to missed diagnoses. However, attempts to examine these regions without an appropriate imaging technique require considerable effort, and tissue structure of lesions may be damaged in the process.

Medical imaging technology has been widely applied in forensic science to detect injuries and pathological changes in determining the cause of death. The combination of PMCT and PMCTA is time conserving and can provide three-dimensional images that enable clear visualization of deep and narrow blood vessels [5, 11, 13, 14]. The combined approach has been applied, and has demonstrated its ability to detect vascular injuries and abnormities including cardiac and aortic injuries, intracranial arteriovenous malformation, and aneurysm [4, 15, 16]. This promising modality has been considered to be an effective supplement to autopsy or an alternative if autopsy is refused [11, 17].

Worldwide, there are several main forms of whole body PMCTA, and many studies have investigated performing PMCTA on a single organ, such as the heart or brain, which represents a highly focused application of the technique [14, 18, 19]. PMCTA techniques are differentiated according to the delivery, type, and targets of the contrast agent. The multiphase PMCTA (MPMCTA) from Switzerland is currently the best evaluated post-mortem imaging method to precisely localize lesions even from very small vessels [11]. This study proposed a new approach to whole-body PMCTA―left ventricle cardiac puncture―which has high success rates and provides adequate visualization of arteries. Image quality is dependent on device performance, scanning parameters, body condition, and performance of device-dependent software and operator skill through digital postprocessing [4]. The PMCTA images obtained in this study were clear. Complete observation of the trunk and branches of the blood vessels could be achieved. Images were with a level of quality satisfying the diagnostic demands for vascular injury and diseases. Also the accuracy of the technique was validated by post-scanning autopsies.

The advantages of this imaging technique over more traditional methods include: simple operation, less time consuming; lower associated costs; no requirement for specialized equipment; and suitable for facilities not equipped with cardiopulmonary bypass machines or other specialized equipment. The heart is a big target and the heart chamber has a large volume, so operators have less to worry about missing or piercing the heart. In addition, this approach could provide heart blood samples for toxicological analysis since in some cases both heart blood and peripheral blood are needed.

Whole-body PMCTA via left ventricle cardiac puncture approach does, however, have limitations. This method is currently only able to visualize the systemic arterial circulatory system. Although imaging the systemic arterial circulatory system is sufficient for the diagnostic demands of the majority of cases, its inability to visualize the coronary arterial system, pulmonary arterial circulatory and venous systems has led to certain constraints. The metal puncture needle would introduce some artifacts during the CT scan, but did not affect the observation and diagnosis. And the artifacts could be completely eliminated by pulling out the needle after perfusion but prior to CT scan. The contrast agent used in the study did not cause artifacts but the agent in the vessels will begin to diffuse into the surrounding soft tissues in a period of time. Judging from the PMCTA images, such diffusion did not bring much impact on the demonstration and diagnosis of vascular abnormalities, and the original state of the soft tissue has been recorded by the previous PMCT scan. Due to restrictions imposed by local laws, customs, and cultures in certain countries and regions (e.g., China), it is difficult to conduct timely autopsies after death, and bodies may need to be cryopreserved for days, months, or even years. Hence, autopsies primarily involve bodies that have been frozen and thawed. Moreover, decomposition and thawing impact the results of angiography to a significant extent. The properties of vessel walls will be affected by putrefaction during the long time preservation, and perfusion of such a fragile venous system would lead to multiple ruptures of small veins and venous plexus (e.g. visceral vessels). In this study, the blood vessels below the aortic arch in cadaver No.10 could not be visualized. This may have been due to incomplete thawing of the body or residual ice in the aortic lumen, which obstructed diffusion of the contrast agent, or blockage from intravascular blood clots. To further explore and refine this imaging approach, subsequent investigations should focus on problems such as eliminating intravascular blood clots and other difficulties associated with venous angiography.

In summary, this study introduced a novel whole-body PMCTA approach, which is simple, less time-consuming, provides adequate image quality, with accuracy of diagnosis and low demands for specialized equipment and funding. This left ventricle cardiac puncture approach is suitable for facilities not equipped with cardiopulmonary bypass machines or other specialized equipment, or available funding for post-mortem vascular examination.

## References

[1] Vander Plaetsen S, De Letter E, Piette M, Van Parys G, Casselman JW, Verstraete K. Post-mortem evaluation of drowning with whole body CT. Forensic science international. 2015;249:35–41. doi: 10.1016/j.forsciint.2015.01.008 25656400 10.1016/j.forsciint.2015.01.008

[2] Schweitzer W Bartsch C, Ruder T, Thali M. Virtopsy approach: structured reporting versus free reporting for pmct findings. Journal of Forensic Radiology & Imaging. 2013;2(1):28–33.

[3] Press C, Approach TV. The Virtopsy Approach. Crc Press 2009.

[4] Zhou S, Wan L, Shao Y, Ying C, Wang Y, Zou D, et al Detection of aortic rupture using post-mortem computed tomography and post-mortem computed tomography angiography by cardiac puncture. International Journal of Legal Medicine. 2016;130(2):469–74. doi: 10.1007/s00414-015-1171-9 25773916 10.1007/s00414-015-1171-9 PMC4767854

[5] Grabherr S, Grimm J, Dominguez A, Vanhaebost J, Mangin P. Advances in post-mortem CT-angiography. British Journal of Radiology. 2014;87(1036):20130488 doi: 10.1259/bjr.20130488 24234582 10.1259/bjr.20130488 PMC4067028

[6] Inokuchi G, Makino Y, Yajima D, Motomura A, Chiba F, Torimitsu S, et al A case of acute subdural hematoma due to ruptured aneurysm detected by postmortem angiography. International Journal of Legal Medicine. 2016;130(2):441 doi: 10.1007/s00414-015-1262-7 26362305 10.1007/s00414-015-1262-7

[7] Hussami M, Grabherr S, Meuli RA, Schmidt S. Severe pelvic injury: vascular lesions detected by ante- and post-mortem contrast medium-enhanced CT and associations with pelvic fractures. Int J Legal Med. 2016 Epub 2016/11/29. doi: 10.1007/s00414-016-1503-4 . 27891547 10.1007/s00414-016-1503-4 PMC5388710

[8] Grabherr S, Doenz F, Steger B, Dirnhofer R, Dominguez A, Sollberger B, et al Multi-phase post-mortem CT angiography: development of a standardized protocol. Deutsche Zeitschrift Für Die Gesamte Gerichtliche Medizin. 2010;125(6):791–802. 10.1007/s00414-010-0526-5 21057803

[9] Grabherr S, Gygax E, Sollberger B, Ross S, Oesterhelweg L, Bolliger S, et al Two-step postmortem angiography with a modified heart-lung machine: preliminary results. Ajr Am J Roentgenol. 2008;190(2):345–51. doi: 10.2214/AJR.07.2261 18212219 10.2214/AJR.07.2261

[10] Sakamoto N, Senoo S, Kamimura Y, Uemura K. Case report: cardiopulmonary arrest on arrival case which underwent contrast-enhanced postmortem CT. J Jpn Assoc Acute Med. 2009;30:114–5.

[11] Grabherr S, Grimm JM, Heinemann A. Atlas of postmortem angiography: Springer; 2016.

[12] Morgan B, Sakamoto N, Shiotani S, Grabherr S. Postmortem computed tomography (PMCT) scanning with angiography (PMCTA): a description of three distinct methods. Essentials of autopsy practice: Springer; 2014 p. 1–21.

[13] Saunders SL, Morgan B, Raj V, Rutty GN. Post-mortem computed tomography angiography: past, present and future. Forensic Science Medicine & Pathology. 2011;7(3):271–7. 10.1007/s12024-010-9208-3 21153718

[14] Rutty GN. Essentials of Autopsy Practice: advances, updates and emerging technologies. Leicester: Springer Science & Business Media; 2013.

[15] Qian H, Shao Y, Li Z, Huang P, Zou D, Liu N, et al Diagnosis of a Cerebral Arteriovenous Malformation Using Isolated Brain Computed Tomography Angiography: Case Report. The American Journal of Forensic Medicine and Pathology. 2016;37(3):201–4. doi: 10.1097/PAF.0000000000000247 27367577 10.1097/PAF.0000000000000247

[16] Michaud K, Grabherr S, Lesta Mdel M, Augsburger M, Doenz F, Mangin P. Ruptured pseudo-aneurysm of a femoral artery in a drug abuser revealed by post-mortem CT angiography. Int J Legal Med. 2013;127(4):819–23. Epub 2012/12/13. doi: 10.1007/s00414-012-0803-6 . 23232543 10.1007/s00414-012-0803-6

[17] Scholing M, Saltzherr TP, Ph FKJ, Ponsen KJ, Reitsma JB, Lameris JS, et al The value of postmortem computed tomography as an alternative for autopsy in trauma victims: a systematic review. European Radiology. 2009;19(10):2333–41. doi: 10.1007/s00330-009-1440-4 19458952 10.1007/s00330-009-1440-4 PMC2758189

[18] Morgan B, Biggs MJ, Barber J, Raj V, Amoroso J, Hollingbury FE, et al Accuracy of targeted post-mortem computed tomography coronary angiography compared to assessment of serial histological sections. Int J Legal Med. 2013;127(4):809–17. doi: 10.1007/s00414-012-0790-7 . 23142905 10.1007/s00414-012-0790-7

[19] Franckenberg S, Schulze C, Bolliger SA, Gascho D, Thali MJ, Flach PM. Postmortem angiography in computed tomography and magnetic resonance imaging in a case of fatal hemorrhage due to an arterio-venous malformation in the brain. Legal Medicine. 2015;17(3):180–3. doi: 10.1016/j.legalmed.2014.11.006 25572321 10.1016/j.legalmed.2014.11.006

